# Weekly Load Variations of Distance-Based Variables in Professional Soccer Players: A Full-Season Study

**DOI:** 10.3390/ijerph17093300

**Published:** 2020-05-09

**Authors:** Filipe Manuel Clemente, Rui Silva, Daniel Castillo, Asier Los Arcos, Bruno Mendes, José Afonso

**Affiliations:** 1 Escola Superior Desporto e Lazer, Instituto Politécnico de Viana do Castelo, Rua Escola Industrial e Comercial de Nun’Álvares, 4900-347 Viana do Castelo, Portugal; rui.s@ipvc.pt; 2 Instituto de Telecomunicações, Delegação da Covilhã, 1049-001 Lisboa, Portugal; 3 Faculty of Health Sciences, Universidad Isabel I, Calle de Fernán González, 76, 09003 Burgos, Spain; danicasti5@gmail.com; 4 Department of Physical Education and Sport, University of the Basque Country, Universidad del País Vasco-Euskal Herriko Unibertsitatea, Lasarte 71, 01007 Vitoria, Gasteiz, Spain; asierlosarcos@gmail.com; 5 Faculty of Human Kinetics, University of Lisboa, Estr. da Costa, Cruz Quebrada, 1495-751 Lisboa, Portugal; brunomendes941982@gmail.com; 6 Centre for Research, Education, Innovation and Intervention in Sport, Faculty of Sport, University of Porto, R. Dr. Plácido da Costa 91, 4200-450 Porto, Portugal; jafonsovolei@hotmail.com

**Keywords:** football, performance, external load, workload, training monotony, training strain

## Abstract

The aim of this study was two-fold: (1) to analyze the variations of acute load, training monotony, and training strain among early (pre-season), mid (first half of season), and end season (second half of season) periods; (2) to compare these training indicators for playing positions in different moments of the season. Nineteen professional players (age: 26.5 ± 4.3 years; experience as professional: 7.5 ± 4.3 years) from a European First League team participated in this study. The players were monitored daily over a 45-week period for the total distance (TD), distance covered (DC) at 14 km/h^−1^ or above (DC > 14 km/h), high-speed running above 19.8 km/h^−1^ (HSR) distance, and number of sprints above 25.2 km/h^−1^. The acute load (sum of load during a week), training monotony (mean of training load during the seven days of the week divided by the standard deviation of the training load of the seven days), and training strain (sum of the training load for all training sessions and matches during a week multiplied by training monotony) workload indices were calculated weekly for each measure and per player. Results revealed that training monotony and training strain for HSR were meaningfully greater in pre-season than in the first half of the in-season (*p* ≤ 0.001; d = 0.883 and *p* ≤ 0.001; d = 0.712, respectively) and greater than the second half of the in-season (*p* ≤ 0.001; d = 0.718 and *p* ≤ 0.001; d = 0.717). The training monotony for the sprints was meaningfully greater in pre-season than in the first half of in-season (*p* < 0.001; d = 0.953) and greater than the second half of in-season (*p* ≤ 0.001; d = 0.916). Comparisons between playing positions revealed that small-to-moderate effect sizes differences mainly for the number of sprints in acute load, training monotony, and training strain. In conclusion, the study revealed that greater acute load, training monotony, and training strain occurred in the pre-season and progressively decreased across the season. Moreover, external defenders and wingers were subjected to meaningfully greater acute load and training strain for HSR and number of sprints during the season compared to the remaining positions.

## 1. Introduction

The training process can be individualized using proper monitoring approaches that help to identify daily variations in a player’s status [1] and the load that he or she is experiencing during training sessions and matches [2]. Training load quantification can also be a good approach for ensuring that core training principles are being followed, namely individualization, progressive overload, and variation [3]. In specific, variation can be crucial for avoiding monotony in training plan. Variation may also provide players with periods of recovery to promote supercompensation and prevent exposure to overreaching [4,5], as this would put the player in a close relationship with overtraining. In the specific case of training load quantification, there are two notable dimensions to be considered [6]: (i) internal load (psychobiological responses to a given level of external load) and (ii) external load (the physical demand imposed by the task/exercise). Training load quantification is included in a player’s monitoring cycle in which both internal and external loads are controlled, as is the player’s well-being and readiness [7]. In the context of team sports, external load has been commonly quantified by using: (i) typical distances covered at different speed thresholds; (ii) events related to changes in velocity (e.g., accelerations, decelerations, and changes in direction); and (iii) events derived from inertial sensors/accelerometers (e.g., player load, impacts, stride variables) [8]. To quantify the external load measures, many researchers have used microelectromechanical systems (MEMS) due to their convenience, validity, and reliability in integrating multidimensional information [9]. The use of these systems aids the daily practice of sports science practitioners in controlling the within- and between-week variations in the load imposed on players. In turn, they can preventively identify the best approaches to use to optimize training. They can also identify injury risks and overreaching [10,11].

Derived from the session loads, some weekly workload indices as training monotony or training strain were introduced to control within-week load variation and the amount of strain occurred in the week, respectively. Monotony is calculated by dividing the daily mean load by the standard deviation [12]. The training strain is the product of weekly training load and training monotony [12]. Both indices were first calculated [12] using the session-rate of perceived exertion (s-RPE), which is calculated by multiplying the score reported on a 10-point scale of exertion by the duration of the training session (in minutes). Higher training monotony scores may suggest low standard deviations of the mean, thus suggesting small variations within a week. Higher training strain suggests greater acute loads imposed with small within-week variations.

The training monotony and training strain workload indices were firstly introduced to monitor overtraining syndrome [12], however some suggestions of high training monotony and training strain levels have been associated with increases in injury risk. In a prospective longitudinal cohort study on Dutch soccer players, in which load, injuries and illnesses were tracked over two seasons, higher injury risks and illnesses increased when training monotony was elevated [13]. However, in other prospective longitudinal cohort study conducted in 130 professional soccer players, it was verified that high levels of training monotony were associated with decreased injury incidences, while a high training strain was associated with greater injury incidences [14]. Studying possible variations between weeks, a study conducted on 45 professional soccer players revealed no meaningful variation in sRPE-based training monotony or training strain over four weeks [15]. Beyond that, in a study conducted on 27 professional soccer players, possible relationships between s-RPE acute load (accumulated load of a week), training monotony, training strain, and variations in fitness status were tested after the pre-season [16]. The authors found that the more meaningful correlations were obtained between acute load and fitness variations.

Although workload indices may help to understand the load dynamics within- and between-weeks it must also use to compare the magnitude of stimulus between different playing positions. In fact, physical demands imposed by the soccer match is quite different from position to position [17]. Thus, and assuming that field-based training sessions are specific and try to keep similarities to real matches, the training load will be also different between playing positions. However, descriptive data regarding different training load measures are not consensual with some studies reporting no external load variations in sessions between positions [18] while others using internal load markers found significant differences between positions [19]. The comparisons of load between playing positions are few and, additionally, this is a lack of research analyzing weekly workload indices (considering that previous studies reported load by each session). To the best of our knowledge, how such indices (acute load, training monotony and training strain) vary across a full-season and according to playing position has not been explored in the literature. Such an exploration could improve our understanding of the dynamics of the workload experienced by professional players. Moreover, we have identified only one article reporting training monotony and training strain using external load measures derived from MEMS data [20]. However, the variation of load and the exposure to high levels of training monotony and training strain may depend on internal and external load measures, thus providing more useful information on the mechanical and physiological demands across a week [20]. 

Therefore, the purpose of this study was two-fold: (1) to analyze the variations of acute load, training monotony and training strain among early (pre-season), mid (first half of season) and end season (second half of season) periods; (2) to compare these training indicators (i.e., acute load, training monotony and training strain) for playing positions in different moments of the season.

## 2. Materials and Methods 

### 2.1. Experimental Approach and Procedures

A descriptive research design was used in this study. Players from a professional team that participated in the First League of a European country (included in the “big five” leagues) were monitored daily. Their external loads for training sessions and matches were measured over a full season. The study began on 3 July 2018 and ended on 9 May 2019. Forty-five weeks were analyzed (Table 1). Each player used an 18 Hz MEMS unit during the training sessions and matches. The external load measures of total distance (TD), distances covered at 14 km/h^−1^ or above (DC > 14 km/h), high-speed running above 19.8 km/h^−1^ (HSR) distance, and number of sprints above 25.2 km/h^−1^ were collected. The acute load (accumulated load of training and matches of each week) was calculated for each player. Moreover, the training monotony (within-week load variation) and training strain (training monotony multiplied by acute load) were calculated once per week for each measure and player. Following the objectives of this study, the acute load, training monotony, and training strain calculations for each external load variable were presented throughout the 45 weeks.

**Figure 1:**
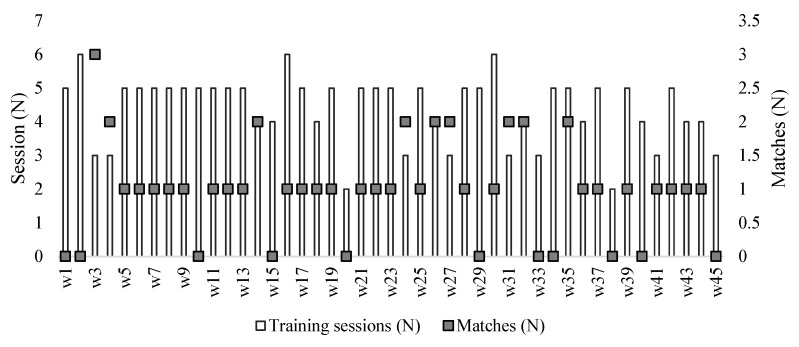
Timeline of the observational study and number of sessions and matches. w: number of the week in the timeline.

The period of collection was organized into three periods: (i) pre-season (PS: week 1 to week 6); (ii) the first half of the season (1st HS: week 6 to week 33); and (iii) the second half of the season (2nd HS: week 34 to week 45). The criteria used to split and organize the periods were as follows: (i) PS (first day of training sessions until the last week without official matches for the First League); (ii) 1st HS (the week that had the first official match until the week that had the last official match of first round of the First League); (iii) 2nd HS (the week that had the first official match of the second round until the week that had the last official match for the First League). Matches for other competitions (e.g., European league matches or national cups) were also included in these periods. The number of training sessions and matches that players participated in each week can be observed in Figure 1. A total of 197 training sessions and 44 matches were monitored. 

The difference in the number of matches between the first and second halves of the season is due to the greater number of matches played in the first half of the season, which is when national cups (matches additional to regular competition) take place. Finally, players included in the sample were categorized into the following groups according to their typical playing position: (i) goalkeeper (GK), (ii) external defender (ED), (iii) central defender (CD), (iv) midfielder (MF), (v) winger (W), and (vi) striker (ST).

### 2.2. Participants

Nineteen professional players (age: 26.5 ± 4.3 years old; body mass: 75.6 ± 9.6 kg; height: 180.2 ± 7.3 cm; experience as professionals: 7.5 ± 4.3 years) from a European First League team participated in this study. From the total number of players, three were ED, four were CD, six were MF, four were W, and two were ST. The following inclusion criteria was defined: (i) players were part of the team from week 1 to week 45; (ii) players were not injured longer than three consecutive weeks; (iii) for each analyzed week, the player participated in all training sessions of that week and, additionally, at least at 15 minutes of a match occurred in such week. The sample size estimation was calculated in GPower software (v3.1.9.7, University of Duesseldorf, Duesseldorf, Germany) for an alpha of 0.5 and beta of 0.8. Results suggested an N of 29 players. However, and due to the limitations of getting this size of the sample in this kind of longitudinal approach in professional soccer players, only nineteen players were analyzed in our study. The players were previously informed about the study design and procedures. After receiving the information, the players signed a free consent about his participation in the study. Despite the monitoring process belonging to the daily routine in the club, they made it voluntarily. The ethical standards of the updated Declaration of Helsinki (2013 version) were ensured during the study. The study was approved by the scientific council of the School of Sport and Leisure (ID: 2018ESDL031). 

### 2.3. External Load Quantification

Each player used the same MEMS unit during the period of the study. The unit consisted in an 18-Hz MEMS, integrating a 100-Hz gyroscope, 100-Hz tri-axial accelerometer and 10-Hz magnetometer (STATSports, Apex, Newry, Northern Ireland). The validity and reliability of MEMS was previously tested revealing good levels of coefficient of variation (<2.3% in all the measures) in the different speed thresholds [21] and excellent inter-unit reliability for peak velocity [22]. The range of satellites during data collection was between 18 and 21. The MEMS units were placed on a specific vest positioning the unit between the scapula. The MEMS-derived data was downloaded and treated by the STATSports Apex software (version 5.0, STATSports, Apex, Newry, Northern Ireland).

The following measures were daily collected (for both, training sessions and matches): (i) TD (consisting in the total distance covered by players, independently from the speed thresholds); (ii) DC≥14 km/h^−1^ (distance covered by the players at speed of 14 km/h^−1^ or above); (iii) DC at HSR (i.e., at a speed of 19.8 km/h^−1^ or above); and (iv) number of times that a speed of 25.2 km/h^−1^ or higher was achieved in running) [21]. The individual external load per each session and match was collected. The AL of each week was calculated per player (sum of the load of all sessions and matches) for the above-mentioned measures. Moreover, training monotony (mean of training load during the seven days of the week divided by the standard deviation of the training load of the seven days) and training strain (sum of the training load for all training sessions and matches during a week multiplied by training monotony) were also calculated for each of the MEMS measures. Based on calculation of acute load, training monotony and training strain, the external load measures were calculated (example for TD: wTD (weekly total distance); mTD (monotony total distance); and sTD (strain total distance)) for further comparisons between periods of the season and playing positions. Within each training period, the mean of weeks occurred in such period were considered for all the measures.

### 2.4. Statistical Procedures

Data was preliminarily tested for outliers, normality and homogeneity. Cases of missing data in periods of one week were considered and treated using the mean of the player for a period of a mesocycle (4-weeks). Results were presented in form of text and figures, as means with standard deviation (SD). After confirmation of assumptions of normality (N > 30, thus assuming the central limit theorem) and homogeneity (Levene; *p* > 0.05), the weekly load (acute load, training monotony, training strain) was compared between periods of the season (PS; 1stHS; 2ndHS) and between playing positions (ED; CD; MF; W; ST) using a repeated measures ANOVA, followed by Tukey HSD post hoc test for pairwise comparisons. Both tests were executed in SPSS (version 25.0, IBM^®^, Chicago, IL, USA) for a *p* ≤ 0.05. The magnitude of differences in pairwise comparisons were tested using the standardized effect size of Cohen (d) for a 95% confidence interval (95% CI). The inference of magnitudes was made using the following thresholds: [0.0; 0.2], trivial; [0.2; 0.6], small; [0.6; 1.2], moderate; [1.2; 2.0], large; >2.0, very large [23].

## 3. Results

Table 1 presents the differences of acute load, training monotony, and training strain for TD, D >14 km/h, HSR, and number of sprints between the PS, 1st HS, and 2nd HS (periods of the season). The training monotony for HSR was meaningfully greater in PS than in the 1st HS (44%; *p* ≤ 0.001; d = 0.883, moderate ES) and greater than the 2nd HS (44%; *p* ≤ 0.001; d = 0.718, moderate ES). Training strain for HSR was meaningfully greater in the PS than in the 1st HS (77%; *p* ≤ 0.001; d = 0.712, moderate ES) and greater than the second half of in-season (84%; *p* ≤ 0.001; d = 0.717, moderate ES). The training monotony of the D >14 km/h were meaningfully greater in PS than in the 2nd HS (58%; *p* ≤ 0.001; d = 0.657, moderate ES) and TA was meaningfully greater in PS than in the 1st HS (102%; *p* ≤ 0.001; d = 0.910, moderate ES) and greater than the 2nd HS (97%, *p* ≤ 0.001; d = 0.765, moderate ES). The training monotony for the number of sprints was meaningfully greater in PS than in the 1st HS (50%; *p* ≤ 0.001; d = 0.953, moderate ES) and greater than the 2nd HS (50%; *p* ≤ 0.001; d = 0.916, moderate ES).

Table 2 presents the differences between playing positions for acute load, training monotony, and training strain for TD. No significant differences (*p* > 0.05) were found between playing positions regarding total distances measure. 

Table 3 presents the differences between playing positions for acute load, training monotony, and training strain for HSR distances. The acute load of HSR was meaningfully greater for wingers than for central defenders (74%; *p* ≤ 0.001; d = 0.685, moderate ES). Training strain was meaningfully greater for external defenders than for central defenders (119%, *p* ≤ 0.001; d = 0.742, moderate ES).

Table 4 presents the differences between playing positions for acute load, training monotony, and training strain for distances covered at 14 km/h or above. The acute load was meaningfully greater for external defenders than for central defenders (65%; *p* ≤ 0.001; d = 0.644, moderate ES). 

Table 5 presents the differences between playing positions for acute load, training monotony, and training strain for the number of sprints. The acute load for the number of sprints were meaningfully greater for wingers than midfielders (146%; *p* ≤ 0.001; d = 1.122, moderate ES) and greater than central defenders (100%; *p* ≤ 0.001; d = 0.831, moderate ES). The external defenders had meaningfully greater acute load than midfielders and greater than central defenders (128%; *p* ≤ 0.001; d = 0.971, moderate ES) and (86%; *p* ≤ 0.001; d = 0.701, moderate ES), respectively. Finally, strikers had meaningfully greater acute load than midfielders (74%; *p* = 0.001; d = 0.670, moderate ES). The training monotony was meaningfully greater for wingers than for midfielders (40%; *p* ≤ 0.001; d = 0.813, moderate ES), and was meaningfully greater for external defenders than midfielders (40%; *p* ≤ 0.001; d = 0.811, moderate ES). The training strain was meaningfully greater for W than for MF (185%; *p* ≤ 0.001; d = 1. 153, moderate ES) and greater than for than central defenders (128%; *p* ≤ 0.001; d = 0.901, moderate ES). Meaningfully greater values were found for external defenders than for midfielders (190%; *p* ≤ 0.001; d = 1.109, moderate ES) and greater than central defenders (129%; *p* ≤ 0.001; d = 0.868, moderate ES). The strikers had meaningfully greater values of training strain than midfielders and central defenders (106%; *p* ≤ 0.001; d = 0.892, moderate ES) and (63%; *p* = 0.010; d = 0.615, moderate ES).

## 4. Discussion

The aims of this study were: (1) to analyze the variations of workload indices between early, mid and end season periods; and (2) to compare these training indicators for playing positions in different moments of the season. The findings revealed meaningful variations of workload indices between season periods and between playing positions. Results revealed no significant differences, although moderate ES differences of TR and training strain for the overall measures between the PS and the 1st HF and 2nd HF were found. The second purpose of our study was to compare acute load, training monotony and training strain for playing positions. Results revealed moderate ES differences mainly for the number of sprints in acute load, training monotony and training strain. Thus, giving coaches new insight about the variation profiling of distance-based MEMS measures.

Regarding the variations of acute load, training monotony and training strain between pre-season, 1st HF and 2nd HF periods, no meaningful differences of acute load, training monotony and training strain for TD were found between periods. Similar findings were found across the in-season blocks in a Spanish elite reserve team [24]. As the first weeks of the season are mainly focused on improving physical condition through augmented volumes of training [25], in the present study there was little variation in acute load for the overall external measures during the first weeks of the season, which is in contrast with a study conducted with Portuguese elite soccer players that found weekly training load variations of 26% of increase to 41% of decrease in the first four weeks of the pre-season [16]. The pattern found in the present study was previously reported in a 45-week study on 30 elite soccer players, in which it was found greater weekly acute load external measures with no significant differences in training load variables between-weeks of the pre-season [26]. The authors also found that the in-season period had no significant differences in acute training load variables, which is congruent with our results that revealed no significant differences in acute load between periods for the overall external measures, although small effect size differences were found for TD ALs between the first and second halves of in-season.

Considering the differences between playing positions the acute load and training strain of HSR was meaningfully greater for wingers than for the remaining positions. Additionally, the acute load of number of sprints were meaningfully greater for external defenders than for central defenders. Among all, these were the unique significant differences found, thus suggesting that, generally, workload indices are not significantly different from position to position. The differences between playing positions on acute distance-based measures has been documented [27,28,29], although those studies only considered defenders, midfielders and attackers for some of distance-based measures. Few studies [30,31,32] considered the five outfield positions (i.e., external defender, central defender, midfielder, winger and striker) for the different intensity zones external measures as applied in the present study. The lack of differences between positions for TD found in the present study are in line with the results found in an observational study that analyzed 30 matches of the Spanish league and champions league [17]. However, they only analyzed the match distances in which players covered ~10.000 m, although the same lack of differences were found when considering weekly sessions of training, showing TD covered between 4.500 m to 7.000 m and HSR distances reaching up to 1.000 m per training session [33], which are approximately the weekly mean values reported in the present study for the TDs, however, it was found greater weekly loads for HSR (up to 2416.7 m) in the present study. 

Little research is known about playing position-dependencies in relation to training monotony and training strain of distance-based measures derived from MEMS. In fact, only one study used this new approach of calculating training monotony and training strain [20]. In that study, it was found that training monotony and training strain revealed trivial effects on training performance of 36 elite Australian footballers, although it was admitted that the variations of those indices were difficult to understand, as it was in the present study. For instance, in the present study, training monotony for TD showed a “w-shape” fluctuating pattern between week 1 to week 30, while for training strain, a pattern of 2 to 4-week mesocycle with relatively low values followed by a sudden increase in the following week was found until the end of the season which may be due to congested weeks. Also, HSR had little training monotony variation from week 6 to week 32, followed by a more accentuated “w-shape” fluctuating pattern from week 33 to 42, while training strain was higher during the pre-season, revealing a pattern of 2 to 5-week mesocycle of lower values, followed by high increases in the following week, throughout the season. 

Our study has some limitations. The size of the sample is one of the main limitations in the present study, as well as the fact that only one team was analyzed. This issue is one of the limitations of longitudinal studies over a full-season in professional contexts. Future studies should include more than one team aiming to increase the generalizability of the findings. Also, we did not include any internal load measure as s-RPE, which is conventionally used to calculate training monotony and training strain [12], however, this was not part of our objectives. Future research is essential to confirm the patterns of such indices found in this study as well as it would be interesting to investigate the training monotony and training strain profiles of accelerometry-based measures over a full season.

Despite limitations, this study was the first, to the best of our knowledge, to analyze the variations of training monotony and training strain between periods of the season as well as between playing positions through distance-based MEMS measures. The main findings of this study were: 1) mainly trivial effect size differences of AL for all distance-based measures between periods of the season were found, while moderate effect size differences of training monotony and training strain for the overall measures between the pre-season and the first and second halves were observed; and 2) no significant differences were found between positions for the overall acute load, although the number of NS presented the greatest differences between positions in acute load, training monotony and training strain. 

## 5. Conclusions

The first purpose of this study was to analyze the variations of acute load, training monotony, and training strain between pre-season, 1st HS, and 2nd HS periods. Results revealed moderate ES differences of training monotony and training strain for the overall measures between the PS and the 1st HF and 2nd HF. The second purpose of our study was to compare acute load, training monotony, and training strain for playing positions. Results revealed moderate ES differences mainly for the number of sprints in acute load, training monotony, and training strain. Thus, giving coaches new insight about the variation profiling of distance-based MEMS measures.

## Figures and Tables

**Table 1:** Descriptive statistics (mean ± SD) of acute load, training monotony, and training strain for the external load measures in the pre-season, 1st half of the season, and 2nd half of the season.

Measure	PS (Mean ± SD)	1st HS (Mean ± SD)	2nd HS (Mean ± SD)	*p*	ES
wTD (m) ^1^	42,873.3 ± 35,145.6	37,869.1 ± 25,752.2	31,877.4 ± 25,068.4	PS vs. 1st HS: 0.273 PS vs. 2nd HS: 0.005 * 1st HF vs. 2nd HS: 0.015 *	PS vs. 1st HS: 0.184 PS vs. 2nd HS: 0.393 ^&^ 1st HF vs. 2nd HS: 0.235 ^&^
mTD (A.U.)	2.3 ± 1.0	2.0 ± 1.1	2.0 ± 1.1	PS vs. 1st HS: 0.045 * PS vs. 2nd HS: 0.136 1st HF vs. 2nd HS: 0.871	PS vs. 1st HS: 0.276 ^&^ PS vs. 2nd HS: 0.279 ^&^ 1st HF vs. 2nd HS: ≤ 0.001
sTD (A.U.)	97,781.2 ± 92,816	64,907.0 ± 68,971.2	61,041.4 ± 68,891.0	PS vs. 1st HS: 0.001 * PS vs. 2nd HS: ≤ 0.001 * 1st HF vs. 2nd HS: 0.786	PS vs. 1st HS: 0.453 ^&^ PS vs. 2nd HS: 0.487 ^&^ 1st HF vs. 2nd HS: 0.056
wHSR (m)	2563.5 ± 1736.1	2050.5 ± 2153.5	1791.2 ± 1556.7	PS vs. 1st HS: 0.084 PS vs. 2nd HS: 0.008 * 1st HF vs. 2nd HS: 0.233	PS vs. 1st HS: 0.244 ^&^ PS vs. 2nd HS: 0.481 ^&^ 1st HF vs. 2nd HS: 0.131
mHSR (A.U.)	1.3 ± 0.7	0. ± 0.4	0.9 ± 0.5	PS vs. 1st HS: ≤ 0.001 * PS vs. 2nd HS: ≤ 0.001 * 1st HF vs. 2nd HS: 0.871	PS vs. 1st HS: 0.883 ^#^ PS vs. 2nd HS: 0.718 ^#^ 1st HF vs. 2nd HS: ≤ 0.001
sHSR (A.U.)	3012.0 ± 2625.0	1701.8 ± 1686.1	1634.8 ± 1617.4	PS vs. 1st HS: ≤ 0.001 * PS vs. 2nd HS: ≤ 0.001 * 1st HF vs. 2nd HS: 0.890	PS vs. 1st HS: 0.712 ^#^ PS vs. 2nd HS: 0.717 ^#^ 1st HF vs. 2nd HS: 0.040
wDC > 14 km/h (m)	8380.2 ± 6754.2	6413.7 ± 5080.9	5740.4 ± 4780.8	PS vs. 1st HS: 0.006 * PS vs. 2nd HS: ≤ 0.001 * 1st HF vs. 2nd HS: 0.245	PS vs. 1st HS: 0.368 ^&^ PS vs. 2nd HS: 0.493 ^&^ 1st HF vs. 2nd HS: 0.135
mDC > 14 km/h (A.U.)	1.9 ± 1.6	1.2 ± 1.2	1.2 ± 0.8	PS vs. 1st HS: ≤ 0.001 * PS vs. 2nd HS: ≤ 0.001 * 1st HF vs. 2nd HS: 0.996	PS vs. 1st HS: 0.554 ^&^ PS vs. 2nd HS: 0.657 ^#^ 1stHF vs. 2nd HS: ≤ 0.001
sDC > 14 km/h (A.U.)	14,464.9 ± 13,203.5	7160.7 ± 6958.5	7358.3 ± 7657.2	PS vs. 1st HS: ≤ 0.001 * PS vs. 2nd HS: ≤ 0.001 * 1st HF vs. 2nd HS: 0.950	PS vs. 1st HS: 0.910 ^#^ PS vs. 2nd HS: 0.765 ^#^ 1st HF vs. 2nd HS: −0.028
wNS (n)	28.9 ± 25.1	28.6 ± 25.0	26.3 ± 25.2	PS vs. 1st HS: 0.995 PS vs. 2nd HS: 0.709 1st HF vs. 2nd HS: 0.492	PS vs. 1st HS: 0.012 PS vs. 2nd HS: 0.103 1st HF vs. 2nd HS: 0.091
mNS (A.U.)	0.9 ± 0.4	0.6 ± 0.3	0.6 ± 0.3	PS vs. 1st HS: ≤ 0.001 * PS vs. 2nd HS: ≤ 0.001 * 1st HF vs. 2nd HS: 0.987	PS vs. 1st HS: 0.953 ^#^ PS vs. 2nd HS: 0.916 ^#^ 1st HF vs. 2nd HS: ≤ 0.001
sNS (A.U.)	29.2 ± 25.2	20.7 ± 19.5	19.5 ± 21.6	PS vs. 1st HS: 0.002 * PS vs. 2nd HS: 0.003 * 1st HF vs. 2nd HS: 0.779	PS vs. 1st HS: 0.417 ^&^ PS vs. 2nd HS: 0.430 ^&^ 1st HF vs. 2nd HS: 0.059

^1^ wTD: weekly total distance; mTD: monotony total distance; sTD: strain total distance; wHSR: weekly high-speed running; mHSR: monotony high-speed running; sHSR: strain high-speed running; wDC >14 km/h: weekly distance covered at 14 km/h^−1^ or above; mDC >14 km/h monotony distance covered at 14 km/h^−1^ or above; sDC >14km/h: strain distance covered at 14 km/h^−1^ or above; wNS: weekly number of sprints; mNS: monotony number of sprints; sNS: strain number of sprints; PS: pre-season period; 1st HS: first half of the season; 2nd HS: second half of the season; A.U.: arbitrary units; * significant at *p* ≤ 0.05; ES: effect size; ^&^: small ES; ^#^: moderate ES.

**Table 2:** Descriptive statistics (mean ± SD) of acute load, training monotony, and training strain for the total distance between playing positions.

Measure	ED (Mean ± SD)	CD (Mean ± SD)	MF (Mean ± SD)	W (Mean ± SD)	ST (Mean ± SD)	*p*	ES
wTD (m) ^1^	37,175.2 ± 26,732.2	36,590.2 ± 25,433.9	37,358.2 ± 29,528.9	34,950.6 ± 25,391.1	37,141.5 ± 24,309.3	ED vs. CD: ≥ 0.999 ED vs. MF: ≥ 0.999 ED vs. W: 0.946 ED vs. ST: ≥ 0.999 CD vs. MF: 0.999 CD vs. W: 0.984 CD vs. ST: ≥ 0.999 MF vs. W: 0.907 MF vs. ST: ≥ 0.999 W vs. ST: 0.975	ED vs. CD: 0.023 ED vs. MF: −0.006 ED vs. W: 0.085 ED vs. ST: 0.001 CD vs. MF: −0.027 CD vs. W: 0.065 CD vs. ST: −0.022 MF vs. W: 0.086 MF vs. ST: 0.008 W vs. ST: −0.088
mTD (A.U.)	2.0 ± 1.1	2.0 ± 1.1	1.2 ± 1.1	2.0 ± 1.1	2.0 ± 1.0	ED vs. CD: ≥ 0.999 ED vs. MF: 0.998 ED vs. W: ≥ 0.999 ED vs. ST: ≥ 0.999 CD vs. MF: 0.997 CD vs. W: ≥ 0.999 CD vs. ST: ≥ 0.999 MF vs. W: 0.995 MF vs. ST: ≥ 0.999 W vs. ST: ≥ 0.999	ED vs. CD: 0.000 ED vs. MF: 0.727 ^#^ ED vs. W: 0.000 ED vs. ST: 0.000 CD vs. MF: 0.727 ^#^ CD vs. W: 0.000 CD vs. ST: 0.000 MF vs. W: −0.727 ^#^ MF vs. ST: −0.744 ^#^ W vs. ST: 0.000
sTD (A.U.)	74,088.1 ± 82,344.7	76,273.4 ± 71,020.7	60,962.8 ± 72,257.2	61,953.4 ± 62,714.6	62,876.5 ± 68,200.2	ED vs. CD: ≥ 0.999 ED vs. MF: 0.395 ED vs. W: 0.566 ED vs. ST: 0.790 CD vs. MF: 0.272 CD vs. W: 0.426 CD vs. ST: 0.676 MF vs. W: ≥ 0.999 MF vs. ST: ≥ 0.999 W vs. ST: ≥ 0.999	ED vs. CD: −0.028 ED vs. MF: 0.172 ED vs. W: 0.166 ED vs. ST: 0.144 CD vs. MF: 0.213 ^&^ CD vs. W: 0.214 ^&^ CD vs. ST: 0.191 MF vs. W: −0.014 MF vs. ST: −0.027 W vs. ST: −0.014

^1^ wTD: weekly total distance; mTD: monotony total distance; sTD: strain total distance; ED: external defender; CD: central defender; MF: midfielder; W: winger; ST: striker; A.U.: arbitrary units; ES: effect size; ^&^: small ES; ^#^: moderate ES.

**Table 3:** Descriptive statistics (mean ± SD) of acute load, training monotony, and training strain for the high-speed running between playing positions.

Measure	ED (Mean ± SD)	CD (Mean ± SD)	MF (Mean ± SD)	W (Mean ± SD)	ST (Mean ± SD)	*p*	ES
wHSR (m) ^1^	2706.2 ± 3025.7	1387.5 ± 1035.8	1780.1 ± 1525.2	2416.7 ± 1829.0	1775.1 ± 1409.2	ED vs. CD: ≤ 0.001 * ED vs. MF: ≤ 0.001 * ED vs. W: 0.658 ED vs. ST: 0.004 * CD vs. MF: 0.295 CD vs. W: ≤ 0.001 * CD vs. ST: 0.592 MF vs. W: 0.011 * MF vs. ST: ≥ 0.999 W vs. ST: 0.105	ED vs. CD: 0.573 ^&^ ED vs. MF: 0.410 ^&^ ED vs. W: 0.116 ED vs. ST: 0.358 ^&^ CD vs. MF: −0.289 ^&^ CD vs. W: −0.685 ^#^ CD vs. ST: −0.328 ^&^ MF vs. W: −0.385 ^&^ MF vs. ST: 0.003 W vs. ST: 0.377 ^&^
mHSR (A.U.)	1.0 ± 0.5	0.9 ± 0.4	0.9 ± 0.5	1.0 ± 0.6	0.9 ± 0.5	ED vs. CD: 0.341 ED vs. MF: 0.127 ED vs. W: 0.970 ED vs. ST: 0.752 CD vs. MF: 0.999 CD vs. W: 0.10 1CD vs. ST: 0.997 MF vs. W: 0.021 * MF vs. ST: 0.974 W vs. ST: 0.429	ED vs. CD: 0.220 ^&^ ED vs. MF: 0.200 ^&^ ED vs. W: ≤ 0.001 ED vs. ST: 0.200 ^&^ CD vs. MF: ≤ 0.001 CD vs. W: −0.195 CD vs. ST: ≤ 0.001 MF vs. W: −0.184 MF vs. ST: ≤ 0.001 W vs. ST: 0.176
sHSR (A.U.)	2535.0 ± 2303.8	1158.7 ± 1168.3	1567.4 ± 1500.7	2019.3 ± 1882.5	1819.7 ± 1884.4	ED vs. CD: ≤ 0.001 * ED vs. MF: ≤ 0.001 * ED vs. W: 0.075 ED vs. ST: 0.028 * CD vs. MF: 0.194 CD vs. W: ≤ 0.001 * CD vs. ST: 0.060 MF vs. W: 0.101 MF vs. ST: 0.809 W vs. ST: 0.925	ED vs. CD: 0.742 ^#^ ED vs. MF: 0.517 ^&^ ED vs. W: 0.245 ^&^ ED vs. ST: 0.329 ^&^ CD vs. MF: −0.296 ^&^ CD vs. W: −0.544 ^&^ CD vs. ST: −0.452 ^&^ MF vs. W: −0.271 ^&^ MF vs. ST: −0.157 W vs. ST: 0.106

^1^ wHSR: weekly high-speed running; mHSR: monotony high-speed running; sHSR: strain high-speed running; ED: external defender; CD: central defender; MF: midfielder; W: winger; ST: striker; A.U.: arbitrary units; * significant at *p* ≤ 0.05; ES: effect size; ^&^: small ES; ^#^: moderate ES.

**Table 4:** Descriptive statistics (mean ± SD) of acute load, training monotony, and training strain for the DC >14km/h between playing positions.

Measure	ED (Mean ± SD)	CD (Mean ± SD)	MF (Mean ± SD)	W (Mean ± SD)	ST (Mean ± SD)	*p*	ES
wDC ^1^ >14 km/h (m)	7471.4 ± 5418.4	4517.0 ± 3442.5	6805.7 ± 5863.2	6881.7 ± 5221.2	5710.5 ± 4750.4	ED vs. CD: ≤ 0.001 * ED vs. MF: 0.711 ED vs. W: 0.841 ED vs. ST: 0.088 CD vs. MF: ≤ 0.001 * CD vs. W: 0.001 * CD vs. ST: 0.449 MF vs. W: 1.000 MF vs. ST: 0.465 W vs. ST: 0.453	ED vs. CD: 0.644 ^#^ ED vs. MF: 0.117 ED vs. W: 0.111 ED vs. ST: 0.338 ^&^ CD vs. MF: −0.452 ^&^ CD vs. W: −0.530 ^&^ CD vs. ST: −0.302 ^&^ MF vs. W: −0.014 MF vs. ST: 0.196 W vs. ST: 0.231 ^&^
mDC > 14 km/h (A.U.)	1.5 ± 1.9	1.2 ± 0.6	1.2 ± 0.7	1.2 ± 0.7	1.3 ± 1.5	ED vs. CD: 0.350 ED vs. MF: 0.127 ED vs. W: 0.375 ED vs. ST: 0.937 CD vs. MF: 0.999 CD vs. W: ≥ 0.999 CD vs. ST: 0.941 MF vs. W: 0.995 MF vs. ST: 0.829 W vs. ST: 0.956	ED vs. CD: 0.208 ED vs. MF: 0.226 ^&^ ED vs. W: 0.209 ^&^ ED vs. ST: 0.113 CD vs. MF: ≤ 0.001 CD vs. W: ≤ 0.001 CD vs. ST: −0.098 MF vs. W: ≤ 0.001 MF vs. ST: −0.103 W vs. ST: −0.096
sDC > 14 km/h (A.U.)	9012.1 ± 9581.2	6297.5 ± 5581.1	8019.8 ± 8632.5	8365.9 ± 8370.7	7133.3 ± 6937.3	ED vs. CD: 0.035 * ED vs. MF: 0.767 ED vs. W: 0.957 ED vs. ST: 0.453 CD vs. MF: 0.284 CD vs. W: 0.192 CD vs. ST: 0.950 MF vs. W: 0.994 MF vs. ST: 0.920 W vs. ST: 0.811	ED vs. CD: 0.341 ^&^ ED vs. MF: 0.110 ED vs. W: 0.072 ED vs. ST: 0.214 ^&^ CD vs. MF: −0.226 ^&^ CD vs. W: −0.288 ^&^ CD vs. ST: −0.137 ^&^ MF vs. W: −0.041 MF vs. ST: 0.108 W vs. ST: 0.156

^1^ wDC >14 km/h: weekly distance covered at 14 km/h^−1^ or above; mDC >14 km/h monotony distance covered at at 14 km/h^−1^ or above; sDC >14 km/h: strain distance covered at 14 km/h^−1^ or above; ED: external defender; CD: central defender; MF: midfielder; W: winger; ST: striker; A.U.: arbitrary units; * significant at *p* ≤ 0.05; ES: effect size; ^&^: small ES; ^#^: moderate ES.

**Table 5:** Descriptive statistics (mean ± SD) of acute load, training monotony, and training strain for the number of sprints between playing positions.

Measure	ED (Mean ± SD)	CD (Mean ± SD)	MF (Mean ± SD)	W (Mean ± SD)	ST (Mean ± SD)	*p*	ES
wNS (n) ^1^	37.7 ± 28.5	20.3 ± 19.8	16.5 ± 15.3	40.6 ± 27.9	28.7 ± 22.4	ED vs. CD: ≤ 0.001 * ED vs. MF: ≤ 0.001 * ED vs. W: 0.797 ED vs. ST: 0.039 * CD vs. MF: 0.528 CD vs. W: ≤ 0.001 * CD vs. ST: 0.076 MF vs. W: ≤ 0.001 * MF vs. ST: 0.001 * W vs. ST: 0.002 *	ED vs. CD: 0.701 ^#^ ED vs. MF: 0.971 ^#^ ED vs. W: −0.103 ED vs. ST: 0.338 ^&^ CD vs. MF: 0.221 ^&^ CD vs. W: −0.831 ^#^ CD vs. ST: −0.405 ^&^ MF vs. W: −1.122 ^#^ MF vs. ST: −0.670 ^#^ W vs. ST: 0.454 ^&^
mNS (A.U.)	0.7 ± 0.3	0.6 ± 0.3	0.5 ± 0.2	0.7 ± 0.3	0.6 ± 0.2	ED vs. CD: 0.018 * ED vs. MF: ≤ 0.001 * ED vs. W: 0.990 ED vs. ST: 0.389 CD vs. MF: 0.123 CD vs. W: 0.004 * CD vs. ST: 0.929 MF vs. W: ≤ 0.001 * MF vs. ST: 0.039 * W vs. ST: 0.198	ED vs. CD: 0.333 ^&^ ED vs. MF: 0.811 ^#^ ED vs. W: ≤ 0.001 ED vs. ST: 0.369 ^&^ CD vs. MF: 0.411 ^&^ CD vs. W: −0.333 ^&^ CD vs. ST: ≤ 0.001 MF vs. W: −0.813 ^#^ MF vs. ST: −0.500 ^&^ W vs. ST: 0.370 ^&^
sNS (A.U.)	31.6 ± 25.8	13.8 ± 11.7	10.9 ± 10.9	31.1 ± 24.0	22.5 ± 17.7	ED vs. CD: ≤ 0.001 * ED vs. MF: ≤ 0.001 * ED vs. W: 0.999 ED vs. ST: 0.005 * CD vs. MF: 0.621 CD vs. W: ≤ 0.001 * CD vs. ST: 0.010 * MF vs. W: ≤ 0.001 * MF vs. ST: ≤ 0.001 * W vs. ST: 0.010 *	ED vs. CD: 0.868 ^#^ ED vs. MF: 1.109 ^#^ ED vs. W: 0.020 ED vs. ST: 0.388 ^&^ CD vs. MF: 0.259 ^&^ CD vs. W: −0.901 ^#^ CD vs. ST: −0.615 ^#^ MF vs. W: −1.153 ^#^ MF vs. ST: −0.892 ^#^ W vs. ST: 0.389 ^&^

^1^ wNS: weekly number of sprints; mNS: monotony number of sprints; sNS: strain number of sprints; ED: external defender; CD: central defender; MF: midfielder; W: winger; ST: striker; A.U.: arbitrary units; * significant at *p* ≤ 0.05; ES: effect size; ^&^: small ES; ^#^: moderate ES.
